# A refresh of the top 10 research priorities in cystic fibrosis

**DOI:** 10.1136/thorax-2023-220100

**Published:** 2023-06-07

**Authors:** Nicola Jane Rowbotham, Sherie Smith, Zoe C Elliott, Belinda Cupid, Lorna J Allen, Katherine Cowan, Lucy Allen, Alan Robert Smyth

**Affiliations:** 1 University of Nottingham School of Medicine, Nottingham, UK; 2 Parent of Children with CF, Nottingham, UK; 3 Cystic Fibrosis Trust, London, UK; 4 James Lind Alliance, Southampton, UK; 5 NIHR Nottingham Biomedical Research Centre, Nottingham, UK

**Keywords:** Cystic Fibrosis

## Abstract

In 2018 we published the James Lind Alliance (JLA) top 10 priorities for clinical research in cystic fibrosis (CF), chosen jointly by the patient and clinical communities. These priorities have led to new research funding. To establish whether priorities have changed with novel modulator therapies, we undertook an online international update through a series of surveys and a workshop. Patients and clinicians (n=1417) chose the refreshed top 10 from 971 new research questions (suggested by patients and clinicians) and 15 questions from 2018. We are working with the international community to promote research based on these refreshed top 10 priorities.

## Introduction

In 2018 we published the top ten research priorities for cystic fibrosis (CF), jointly developed by the CF patient and clinical communities.^1^ These attracted global interest and have led to new research in CF which focuses on the concerns of patients. Funders have adopted these priorities leading to at least £23 million of UK funding alone.^2^ Much has changed in CF over the past 5 years, with the CF transmembrane conductance regulator (CFTR) modulator therapy available to many (but not all) people with CF (pwCF). The COVID-19 pandemic has also brought changes, with clinical care and research visits taking place virtually. Changes in research delivery may also affect research priorities. It was therefore timely to refresh the top ten priorities for clinical research in CF to ensure that they remain relevant to the CF community.

The original top ten priorities were produced by working with the NIHR-supported James Lind Alliance (JLA) who facilitate priority setting partnerships (PSPs) using methodology which brings patients, carers and clinicians together to prioritise research questions. We were the first PSP to undertake a refresh of disease-specific research priorities and have worked with the support of the JLA Lab – an exploratory space that supports innovation.^3^ Here we describe our methodology and results.

## Methods

Our PSP refresh took place October 2021 – November 2022. We published our protocol before commencing.^4^ As no PSP refresh had occurred previously, we created novel methodology adapting standard JLA methods for priority setting.^5^ We appointed a steering group, representative of the international CF community, for oversight and a smaller management group, for operational delivery.

From January to February 2022, we conducted the first of two online surveys (Survey Monkey). The elicitation survey requested respondent demographics and asked participants to select their top three priorities from the previous top twenty questions. Respondents were also asked to submit up to two new priorities. Surveys were advertised through professional and charity groups and our bespoke Twitter account (@questionCF). Both surveys were available in multiple languages. In a change to our original PSP, non-industry researchers were also invited to participate in the refresh.

Two researchers (NR and SS) independently reviewed all submissions with adjudication by the steering and management groups. Responses which did not include a question and out of scope questions were removed. Where several questions addressed the same issue, they were consolidated into a single “umbrella question”. Any question already answered in the scientific literature was removed.^6^

The standardised umbrella questions were then taken forward to the prioritisation survey (September – October 2022). This allowed respondents to rank their top ten questions. Cumulative scores were calculated for each question and the questions ranked in priority order for the following four groups:

- pwCF
- The subgroup of pwCF who currently do not have access to CFTR modulators
- Friends and family of pwCF
- Healthcare professionals and researchers

Questions in the top ten priorities for each of the three main groups, plus the top five for the sub-group of pwCF not on CFTR modulators (online supplemental 1), were then discussed at an online workshop in November 2022, facilitated by the JLA. Lay and professional participants (recruited from people who completed a widely advertised expression of interest form) selected the final refreshed top ten research priorities. The process involved small group discussion and plenary voting, with all participants having an equal voice.

10.1136/thorax-2023-220100.supp1 Supplementary data

## Results

We had 1608 responses from 1370 respondents to the elicitation survey (59 responses were ‘empty’ and 179 respondents completed the survey more than once). An additional 971 questions were submitted. table 1 shows the demographics of respondents. There were 493 (36%) respondents with CF, 515 (38%) family and friends and 362 (26%) healthcare professionals and researchers. Just over 45% were from the UK, with submissions from 29 countries in total.

**Table 1:** Demographic characteristics of respondents to survey 1 and survey 2

	Survey 1 (n=1370)	Survey 2 (n=1417)
Category of respondent		
People with CF	493 (36%)	477 (34%)
Family & Friends	515 (38%)	522 (37%)
Parent of person with CF	353	404
Spouses or partners	29	13
Other family & friends	133	105
Professionals	362 (26%)	418 (29%)
Medical Doctor	83	111
Non-industry Researcher	76	94
Physiotherapist	61	49
Dietician	45	25
Nurse	37	50
Psychologist	19	17
Pharmacist	15	24
Social Worker	9	11
Clinical trial coordinator	4	10
Other	13	21
Unknown	0	6
Country		
UK	619 (45%)	515 (36%)
USA & Canada	83 (6%)	210 (15%)
Rest of Europe	531 (39%)	536 (38%)
Australia & New Zealand	59 (4%)	69 (5%)
Other	10 (1%)	34 (2%)
Unknown	68 (5%)	53 (4%)
Demographics of respondent with CF (or the family member with CF)		
Age (years)	27 (0–79)	26 (0–80)
Gender	(n=955)	(n=948)
Male	397 (42%)	384 (41%)
Female	539 (56%)	561 (59%)
Other	19 (2%)	3 (0%)
Taking Modulators?	(n=948)	(n=951)
Yes	613 (64%)	590 (62%)
No	197 (21%)	192 (20%)
Not Yet eligible	138 (15%)	169 (18%)

Data are presented as number count (%) or median (range). Denominators are expressed as “n=x” where they vary from the total number of survey respondents.

**Figure 1:**
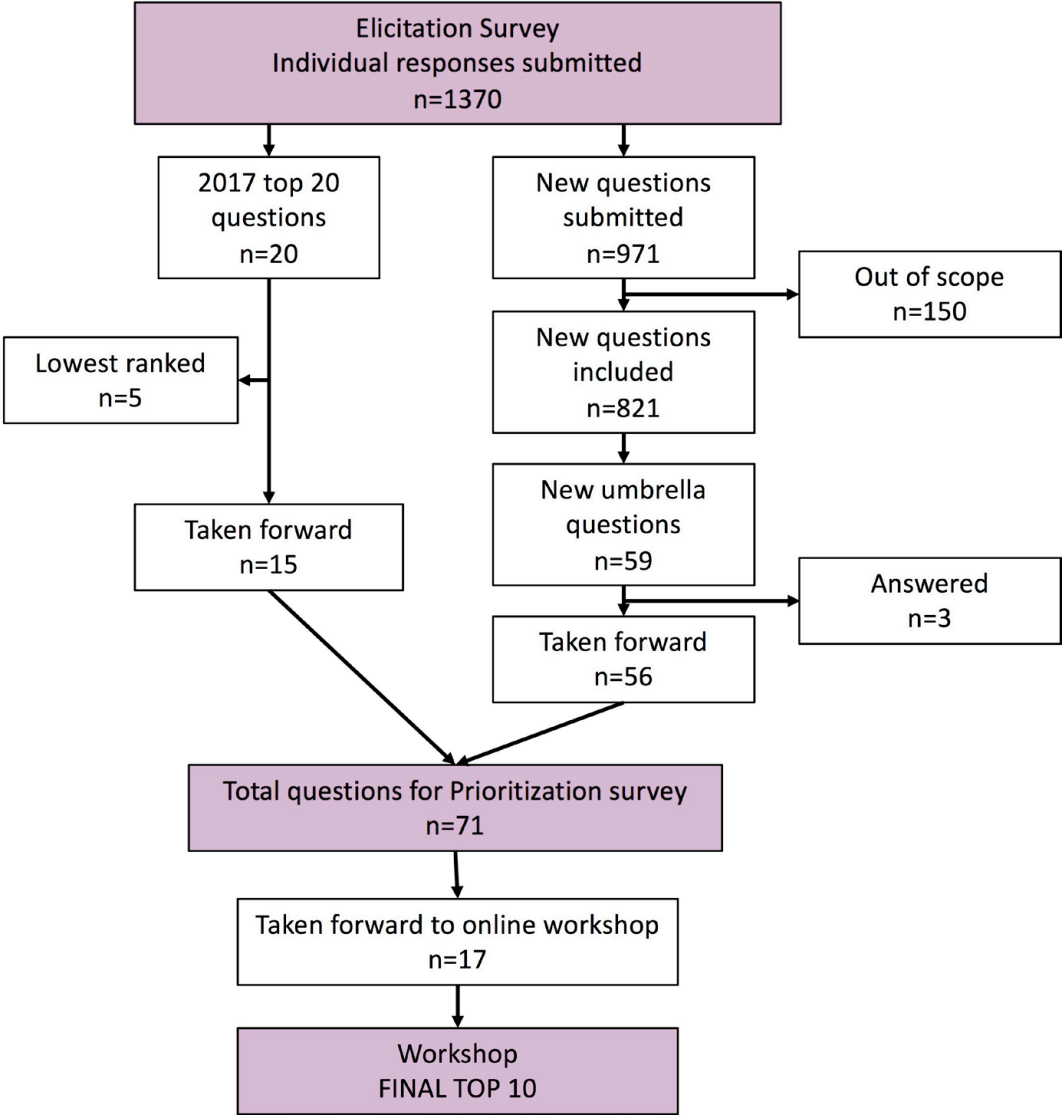
Flow chart showing question processing at the different stages of the James Lind Alliance Priority Setting Partnership refresh in CF.

**Figure 2:**
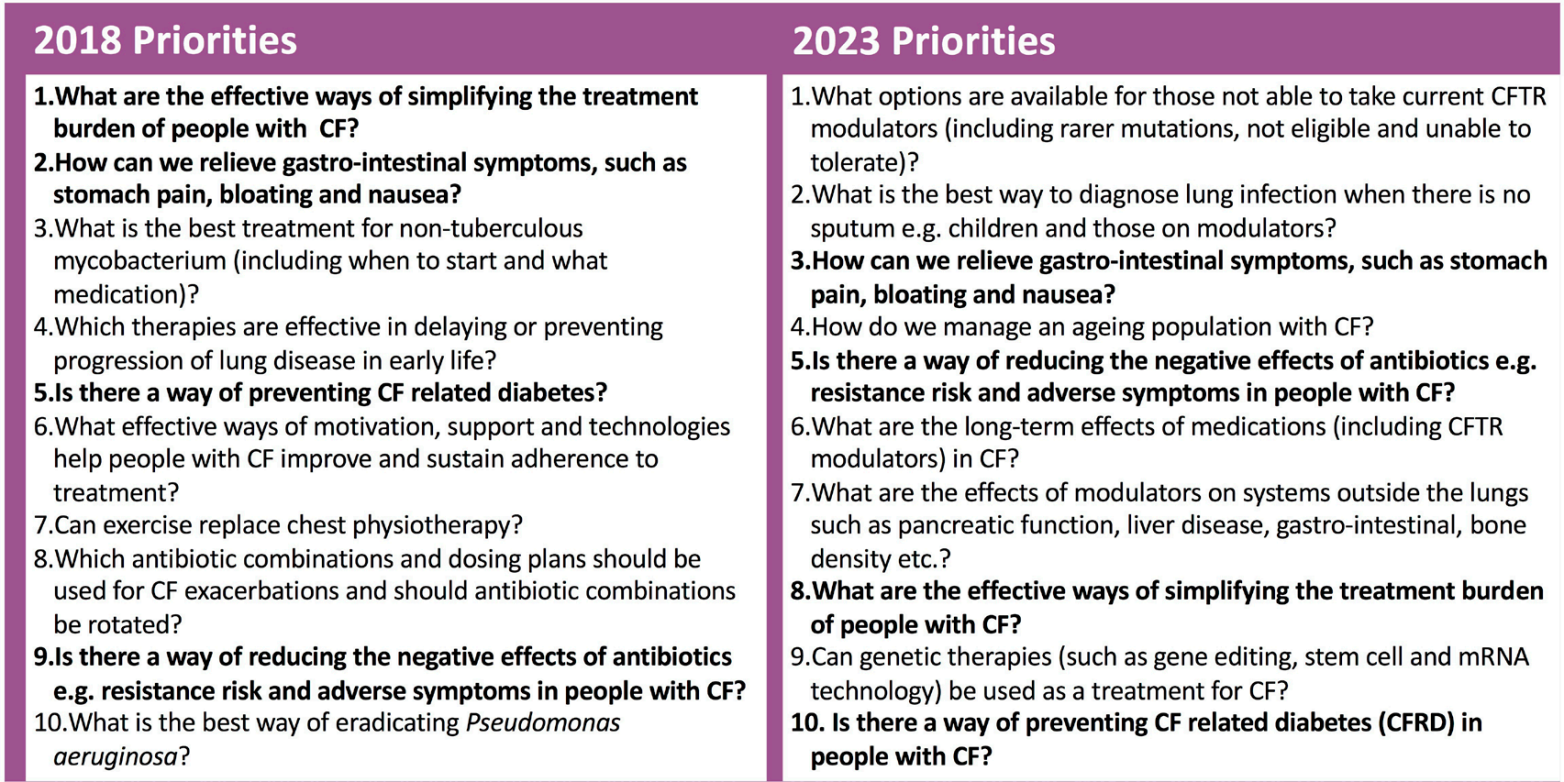
The top 10 questions for research in cystic fibrosis in 2018 and 2023. Those marked in bold feature in both.

Figure 1 describes how the new and existing questions were sorted and refined. There were 821 “in scope” questions which were combined into 59 new umbrella questions. After the checking process three were removed (as they have already been answered) leaving 56 new questions. These were added to the top 15 questions from the previous top 20 and 71 questions were taken to the prioritisation survey. We had 1583 responses to the prioritisation survey (survey 2), completed by 1417 individual respondents, once empty and multiple responses were removed (see table 1 for demographics). The final top 10 questions agreed at the online workshop are shown in figure 2.

## Discussion

This is the first refresh exercise to update the JLA top 10 priorities for a specific medical condition. Our methodology ensured equal participation from people with CF, their family and friends and the clinical/research communities. We have produced a refreshed top 10 list of research questions, which should guide researchers and funders. Four priorities were identified in the original PSP and remain priorities. Six priorities were newly identified by the PSP refresh.

A strength of our study is its international reach. We had a large number of respondents, from countries across the world. While CF is predominantly a disease of Northern European descent, recent prevalence studies show an increase in countries where it was not previously reported.^7^ We acknowledge that our respondents may not be truly representative of the global community as it stands. Data from the UK registry (for 2021) indicate that approximately 32% of pwCF are not currently taking CFTR modulators. In our surveys 36% (elicitation) and 38% (prioritisation) of respondents (or the affected family member) were not taking a CFTR modulator. We adapted the JLA methodology to ensure the top five priorities, for this important sub-group of pwCF, were included in the 17 questions going to the final workshop to choose the top 10.

With the arrival of transformational therapies, available to most (but not all) pwCF, it is vital that the research priorities of funders and researchers remain aligned with those of the patient and clinical communities. Publication of the previous JLA top 10 priorities for CF has led to international funding (via NIHR, CF Trust and CF Foundation for example) for many clinical studies designed to address these priorities.^2^

The previous PSP led directly to the CF-STORM trial (ongoing, ISRCTN14081521) which was developed in response to Question 1 (“What are the effective ways of simplifying the treatment burden of people with Cystic Fibrosis?”) and to the GIFT-CF research programme^8^ designed to start addressing Question 2 (How can we relieve gastro-intestinal symptoms such as stomach pain, bloating and nausea?). Our refreshed JLA top 10 highlights a shift towards longer term priorities and maintenance of health and offers a similar opportunity to the CF research community and their funders to deliver new research addressing these patient and clinician priorities.
