# Supplementary material for: A refresh of the top 10 research priorities in cystic fibrosis
Source: Thorax. 2023 Jun 7;78(8):840–3. doi: 10.1136/thorax-2023-220100 (PMC10359564; doi:10.1136/thorax-2023-220100)
Supplement: Supplementary data [file thorax-2023-220100supp001.pdf]

Group rank from 2nd survey	People with CF	Family & Friends	Health care professionals & researchers	People with CF not on modulator therapy
1	What are the long-term effects of CFTR modulators?*	Can genetic therapies (such as gene editing, stem cell and mRNA technology) be used as a treatment for CF?	What are the long-term effects of CFTR modulators?*	Are options available for those not able to take current CFTR modulators (including rarer mutations, not eligible and unable to tolerate)?
2	Are people with CF at higher risk of certain cancers and what is the best way to detect and manage cancers in people with CF?	What are the long-term effects of CFTR modulators?*	Are options available for those not able to take current CFTR modulators (including rarer mutations, not eligible and unable to tolerate)?	Can genetic therapies (such as gene editing, stem cell and mRNA technology) be used as a treatment for CF?
3	Are options available for those not able to take current CFTR modulators (including rarer mutations, not eligible and unable to tolerate)?	Are options available for those not able to take current CFTR modulators (including rarer mutations, not eligible and unable to tolerate)?	What are the effective ways of simplifying the treatment burden of people with CF?	How best to manage and support people with CF post-transplantation (e.g. mental health, rejection, modulator use)?
4	Can genetic therapies (such as gene editing, stem cell and mRNA technology) be used as a treatment for CF?	What are the effects of modulators on systems outside the lungs such as pancreatic function, liver disease, gastrointestinal, bone density etc?	Can genetic therapies (such as gene editing, stem cell and mRNA technology) be used as a treatment for CF?	How to improve breathing capacity/lung volume?
5	What are the effects of modulators on systems outside the lungs such as pancreatic function, liver disease, gastrointestinal, bone density etc?	What is the optimum treatment regimen for eradication of Pseudomonas in people with CF?	What is the best way to diagnose lung infection when there is no sputum e.g. children and those on modulators?	Is there a way of reducing the negative effects of antibiotics e.g. resistance risk and adverse symptoms in people with CF?
6	How do we manage an ageing population with CF?	What are the effective ways of simplifying the treatment burden of people with CF?	What are the effects of modulators on systems outside the lungs such as pancreatic function, liver disease, gastrointestinal, bone density etc.?	
7	How can we recognise and manage the side-effects of CFTR modulators (including those at greater risk e.g. liver disease)?	Is there a way of preventing CF related diabetes (CFRD) in people with CF?	How do we manage an ageing population with CF?	
8	How can we relieve gastro-intestinal symptoms, such as stomach pain, bloating and nausea?	How can we recognise and manage the side-effects of CFTR modulators (including those at greater risk e.g. liver disease)?	What effective ways of motivation, support and technologies help people with CF improve and sustain adherence to treatment?	
9	Is there a way of preventing CF related diabetes (CFRD) in people with CF?	How can we relieve gastro-intestinal symptoms, such as stomach pain, bloating and nausea?	Is there a way of preventing CF related diabetes (CFRD) in people with CF?	
10	How to improve breathing capacity/lung volume?	What are the long-term effects of medications for CF?*	Can exercise replace chest physiotherapy in people with CF?	

Supplement 1: Table showing the questions taken forward from the ranking survey to the final workshop for each group. * marks questions that were merged into a new question “What are the long-term effects of medications (including CFTR modulators) in CF?” on management group consensus as felt to be too similar.
